# Editorial: Neural bases of reading acquisition and reading disability

**DOI:** 10.3389/fnins.2023.1147156

**Published:** 2023-06-16

**Authors:** Li Hai Tan, Charles A. Perfetti, Johannes C. Ziegler, Bruce McCandliss

**Affiliations:** ^1^Center for Language and Brain, Shenzhen Institute of Neuroscience, Shenzhen, China; ^2^Guangdong-Hongkong-Macau Institute of Central Nerve System (CNS) Regeneration, Jinan University, Shenzhen, China; ^3^Learning Research and Development Center, University of Pittsburgh, Pittsburgh, PA, United States; ^4^Laboratoire de Psychologie Cognitive, Aix-Marseille Université and Centre National de la Recherche Scientifique, Marseille, France; ^5^Graduate School of Education, Stanford University, Stanford, CA, United States

**Keywords:** brain, reading development, reading disability, dyslexia, cross-language

## Introduction

Reading is an essential skill, necessary not only for success in school, but for maintaining a high quality of life in increasingly literate societies. Changes in technology have altered reading formats and increased the range and complexity of literacy contexts, thus placing even more pressure on foundational reading skills. The development of these skills occurs with corresponding neural development associated with learning the forms and functions of written language and their relation to spoken language. Difficulties in learning to read are associated with neural patterns that differ from those of successful learning. Thus, studies of the neural bases of reading inform the development of literacy and reading disability.

Recent progress builds on the remarkable foundation provided by more than 40 years of research from both behavioral and brain studies (Perfetti and Helder, 2022 is a review of some of this research). This foundation established basic facts about the cognitive processes of reading and learning to read, including the acquisition of orthographic, phonological, and semantic information that comprises the identity of printed words, supported by spoken language and conceptual knowledge.

One robust finding is that phonological knowledge, e.g., awareness of meaningless speech segments affects and predicts reading acquisition and developmental dyslexia. Awareness of the fine-grain level of phonology, the phoneme, is especially important for reading alphabetic writing systems and failures to achieve this awareness may be an indicator of risk for dyslexia.

World-wide, most children learn to read a non-alphabetic language. Thus, for understanding both the universality of reading development and its variation with specific languages and writing systems, cross-language research is important. Chinese reading has received the most research attention and can serve as a comparison with alphabetic reading. For instance, phonological knowledge is associated with Chinese reading development as it is with alphabetic reading development. However, visual-orthographic knowledge, visuo-motor, morphological awareness, vocabulary size, working memory, and some other factors may be as important as phonological knowledge in Chinese children's literacy acquisition. The visual complexity of the Chinese character, its monosyllabic mapping, and its (limited) ideographic characteristics, require language-specific features to be part of any universal theory of reading disability.

With this background, this Research Topic brings new research and new reviews of research on the neurobiology of reading ability and disability across languages and writing systems. The 14 articles include 4 reviews and 10 articles of original research across a variety of methods (behavioral, fMRI, ERPs, FRPs, and computational modeling) and languages (English, German, Dutch, French, Finnish, and Chinese). These papers contribute significantly to important issues in reading development and dyslexia, as we discuss below.

## The development of reading skill

### Developing print expertise

In learning to read, children acquire the graphic forms of their writing system, converting them to orthographic units that connect to spoken language. With learning, they acquire precise connections to familiar and whole word orthographic patterns, allowing word identification to shift from computation to memory-based retrieval—fluent word reading. Verhoeven et al. studied the development of this “print tuning” process in learning to read Dutch. They found that fluent word reading did not emerge until children attained a threshold level in decoding accuracy, demonstrating a transition to word reading based on whole word orthographic input.

Complementing this behavioral indicator of print expertise is an ERP component (the N170) measured during word reading. A review by Amora et al. concludes that the distribution of the N170 across left and right hemispheres distinguishes better readers from less able readers and thus serves as an indicator of print expertise.

Developing print expertise involves the interconnections of visual and language brain systems (in occipitotemporal, temporoparietal, and inferior frontal cortex) that support orthographic, phonological, and semantic processing. Reading development must be accompanied by increased connectivity along pathways that implement these processes, e.g., the pathway between the dorsal inferior frontal gyrus and the posterior superior temporal gyrus. In an analysis of fMRI data of 91 native English-speaking children, Wagley and Booth found that, during phonological processing, connectivity along this pathway was related to word reading skill. This result highlights both the functionality of phonological processing along the dorsal pathway during word reading and its relation to actual word reading skill.

If brain organization changes with the development of reading, what happens when the brain has already been organized for reading one language and then acquires a second language? The paper by Cao et al. found that while brain areas active during reading are similar for bilingual readers (L1 Chinese and L2 English), the similarity is greater for adults than children. This is consistent with the convergence hypothesis that increasing proficiency in L2 leads to convergence with the L1 brain network.

Although research attention has focused on cortical areas, subcortical brain areas are also functional in reading through their pathways to cortical areas. In a resting imaging study of 334 Chinese children ages 6–12, Wang et al. found evidence for two different cortico-subcortical pathways, the thalamo-occipital and fronto-striatal circuits. The activation of the thalamus pathway was predicted by reading ability, especially among the younger children. The striatal pathway, perhaps reflecting attention and memory functions, showed a relation that increased with age and became reciprocal. For both younger and older children, reading ability predicted later striatum activation and this association was stronger for older children. For older children only, striatum activation also predicted later reading ability.

A fundamental aspect of typical reading is its dependence on spoken language. This is why phonological processes are so important in the development of reading skill and why spoken language components continue to be present as print expertise develops. In a study of Finnish 12–13-year-old children, Azaiez et al. used a mix of auditory ERP tasks and co-registered eye-tracking/ERPs to provide new insights into the speech-reading relationship. In particular, their study found a correlation between activation in the visual word form area and the superior temporal area (an auditory/speech area) during reading.

### Predicting literacy prior to schooling

Predicting a child's reading success prior to schooling is valuable both theoretically and practically. Beyer et al. report a novel study using machine learning applied to behavioral and fMRI measures taken 2 years prior to reading instruction to predict reading outcomes during the 1st year of schooling. They found that future literacy could be predicted by gray matter volume in the left occipito-temporal cortex and local gyrification in the left insular, inferior frontal, and supramarginal gyri. Behaviorally, phonological awareness was also predictive. Thus, the status of the large-scale reading network at a preliterate age can predict how well children learn to read.

A key factor in reading development is a child's language experience. A young child's early language experience can be captured in their conversation with others, especially parents. A longitudinal study by Weiss et al. demonstrates a positive relationship between measures of parental language input during late infancy and the emergence of literacy skills at age 5. They further report that this relationship is probably mediated by the myelination of the left dorsal pathways of the left hemisphere's emerging language network.

## Dyslexia: causes and interventions

Problems in phonological processing are a primary causal factor in dyslexia. Some proposals have attempted to explain the phonological deficit itself, and thus provide a deeper explanation of dyslexia. One is the neural noise hypothesis (Hancock et al., 2017). “Neural noise” captures the neural response variability that occurs across repetitions of a specific input to an individual: more variability, more noise. On this hypothesis, too much noise interferes with establishing stable representations of linguistic input. However, Tan et al. well-designed a study that found no evidence that dyslexic readers have noisier neural representations than typical readers.

Another proposal is to explain phonological deficits via visual system problems, although assigning a prominent role for visual system deficits has been controversial. Stein's review paper departs from viewing phonological deficits vs. visual deficits as an either-or dichotomy. It argues that a specific visual “transient” process involving magnocellular neurons in the visual system may be a cause of the phonological deficit, especially with the addition of deficits in processing transient auditory signals.

Attempts to explain phonological deficit by linking them to general neural mechanisms are likely to continue. For now, the state of the knowledge is that phonological processing difficulties are the primary causal factor for basic reading problems.

Research on associations of dyslexia with non-phonological abilities is important in gaining a fuller picture of dyslexia. For example, handwriting has been especially strongly associated with reading development in Chinese (Tan et al., 2005) and handwriting problems are often part of the dyslexia profile. Liu et al. add to this picture with their finding that Chinese children with dyslexia showed reduced connectivity between the sensory-motor network and the visual network during handwriting but not drawing.

### Interventions

Well-targeted interventions improve the reading of children and adults with dyslexia. Such improvements are expected to produce neural changes that reflect some degree of “rewiring.” However, two papers in the Research Topic conclude that finding specific brain changes, at least at the group level, is elusive. In their review paper, Braid and Richlan, while noting increased activity in RH homologs to the LH reading network, conclude that neuroplasticity effects do not emerge consistently following a successful intervention.

This conclusion is echoed in an original research paper by Krafnick et al. Their intervention raised reading scores, but without producing changes detectable in fMRI. Interestingly, however, reading gains were predicted by pre-intervention brain activity in bilateral supramarginal/angular gyri (and not predicted by pre-intervention behavioral assessments). Both papers emphasise the value of focusing on individual comparisons rather than group data in looking for brain changes.

Finally, although dyslexia is a human condition, Galaburda's review paper argues that animal models make a contribution to the study of its underlying mechanisms. Developmental cortical anomalies, cerebral asymmetries, functional lateralization, sound processing, and visual perception all can be modeled and genetic contributions to cell functions can be studied in animals.

## Conclusion

We conclude with the suggestion that, by linking brain studies to behavioral indicators, the multi-method, multi-language approaches represented in these papers, add to knowledge about the development of typical reading and the characterizations of reading problems. They also pose new challenges and leave much to be informed by future research.

## Author contributions

All authors listed have made a substantial, direct, and intellectual contribution to the work and approved it for publication.
